# Marker assisted pyramiding of two brown planthopper resistance genes, *Bph3* and *Bph27 (t),* into elite rice Cultivars

**DOI:** 10.1186/s12284-016-0096-3

**Published:** 2016-05-31

**Authors:** Yanling Liu, Liangming Chen, Yuqiang Liu, Huimin Dai, Jun He, Haiyan Kang, Gen Pan, Jie Huang, Zeyu Qiu, Qi Wang, Jinlong Hu, Linglong Liu, Yezhi Chen, Xianian Cheng, Ling Jiang, Jianmin Wan

**Affiliations:** National Key Laboratory of Crop Genetics and Germplasm Enhancement, Nanjing Agricultural University, Nanjing, 210095 People’s Republic of China; Chinese Academy of Agricultural Sciences, Beijing, 100081 People’s Republic of China

**Keywords:** Marker-Assisted selection (MAS), Pyramiding breeding, Brown planthopper, Rice

## Abstract

**Background:**

Brown planthopper (BPH) is the most destructive insect in rice production. Breeding of resistant cultivars is the most cost-effective and environment-friendly strategy for BPH management; however, resistant cultivars are currently hampered by the rapid breakdown of BPH resistance. Thus, there is an urgent need to use more effective BPH resistance genes or pyramiding different resistance genes to develop more durable resistant rice cultivars.

**Results:**

Here a dominant BPH resistance gene *Bph27(t)* were introgressed into a susceptible commercial *japonica* variety Ningjing3 (NJ3) and *indica* variety 93-11 using marker-assisted selection (MAS), respectively. Further, *Bph27(t)* and a durable BPH resistance gene *Bph3* was pyramided by intercrossing single-gene introgressed lines through MAS. The introgression of *BPH* resistance genes significantly improved the BPH resistance and reduced the yield loss caused by BPH.

**Conclusion:**

The development of single and two genes pyramided lines in this study provides innovative resources for molecular breeding of durable BPH-resistant rice cultivars and BPH management through resistant cultivars.

**Electronic supplementary material:**

The online version of this article (doi:10.1186/s12284-016-0096-3) contains supplementary material, which is available to authorized users.

## Background

Rice is a staple food for over half of world population, while serious yield loss was caused by diseases and insects attack annually (Akhtar et al. 2010; Zhai 2011; Hu et al. 2014). Brown planthopper (BPH) has become a major destructive insect and caused seriously loss in rice production (Way and Heong 1994; Heinrichs and Mochida 1984). Nowadays, pesticide abuse in BPH management has heavily polluted the environment and resulted in the tolerance of BPH to the insecticides (Tanaka et al. 2000). Development of resistant rice cultivars is considered the most economic effective and environment-friendly measure for controlling this insect.

In response to the serious damage caused by BPH, resistance varieties were developed sequentially based on the identification of some BPH resistance genes (Suh et al. 2011). However, some of the varieties bearing the BPH resistance genes were quickly broken down within a few years due to rapid adaptation of BPH (Khush 1977). Resistant varieties with *Bph1* were initially released in 1973, but these varieties were already adapted and overcome by BPH in 1975 (Cohen et al. 1997). Then some varieties with the *bph2* derived from ASD7 were released, but again, in the early 1980s, these varieties were also adapted by BPH. Since 1982, some varieties with resistance gene *Bph3* have been released (Khush and Virk 2005). *Bph3* display broad spectrum resistance to all four BPH biotypes. In contrast with *Bph1* and *bph2*, though the varieties harboring *Bph3* have been deployed over 30 years in the Philippines, they still retain effect against BPH (Cruz et al. 2011). Thus, *Bph3* has great potential in developing broad spectrum and durable resistant varieties against BPH. Recently, *Bph3* from Rathu Heenati (RH) has been successfully cloned, which proved that the *Bph3* resistance is conferred by a cluster of three plasma membrane-localized lectin receptor kinases (Liu et al. 2015).

In addition to application of durable resistance genes, pyramiding multiple BPH resistance genes is another efficient strategy to develop durable resistant varieties against BPH. Previously the BPH resistance cultivars were mainly bred by traditional breeding approach, which generally led to time- and labor-consuming and low efficiency, especially for pyramiding multiple genes. Molecular marker-assisted selection (MAS) is considered as an efficient and rapid approach for introducing and pyramiding BPH resistance genes (Qiu et al. 2010). Molecular mapping would facilitate the MAS for providing the resistance genes (Huang et al. 2013; Jena and Kim 2010). So far, 29 BPH resistance genes have been identified from cultivated and wild rice (Cheng et al. 2013; Wu et al. 2014). Among them, previous studies demonstrated that three varieties Balamawee (Ba), Kaharamana and Pokkali might carry the same BPH resistance gene, designed as *Bph9* (Nemoto et al. 1989). Subsequently, Murata et al. and Su et al. located *Bph9* on the long arm of rice chromosome 12 using Pokkali and Kaharamana, respectively (Murata et al. 2000; Su 2006). However, comparing various characteristics of BPH behavior found that the feeding behavior of BPH on Ba was significantly different from that on the other two cultivars. The resistance gene in Ba was then finely mapped on chromosome 4 and named as *Bph27(t)* (He et al. 2013).

In present study, we introgressed *Bph27(t)* into susceptible commercial *japonica* variety Ningjing3 (NJ3) and *indica* variety 93-11 using marker-assisted selection (MAS), respectively. The introgression of *Bph27(t)* significantly improved the BPH resistance level and reduced the yield loss caused by BPH. Further, we pyramided *Bph3* and *Bph27(t)* by intercrossing *Bph3* and *Bph27(t)* single gene introgressed *japonica* lines through MAS. The development of BPH resistance lines with single and two genes pyramided have a promising future in molecular breeding of durable BPH-resistant rice cultivars.

## Results

### Introgression of *Bph27(t)* into *japonica* and *indica* cultivar rice

**Fig. 1:**
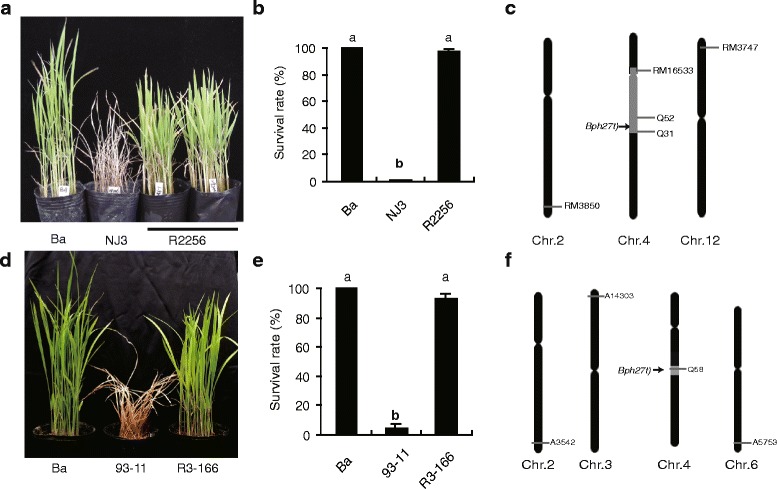
Improvement of BPH resistance by introgression of *Bph27(t)* into *japonica* (**a**-**c**) and *indica* rice cultivars (**d**-**f**), respectively. Letters indicate a significant difference at *P* < 0.01 by the Student’s *t*-test (**b** and **e**). A diagram (**c** and **d**) showing the chromosomal contribution of Ba (gray) and Ningjing 3 or 93-11 (black)

Ningjing3 (NJ3) is one of the most widely cultured elite *japonica* varieties in Jiangsu Providence, China, but it showed highly susceptible to BPH. In order to develop BPH resistant *Japonica* cultivars, NJ3 was used as the recurrent parent to backcross with Ba for six generations and then self-crossed to produce a BC_6_F_2_ population. Two markers (Q52 and Q31) reported by He et al. (2013) were used for tracking introgression of *Bph27(t)* (Table 1). To introgress *Bph27(t)* into *indica* cultivar 93-11, the same procedure as described above was conducted, except that markers were replaced by RM471 and Q58 (Table 1). Finally, a *Bph27(t)*-carrying *japonica* line R2256 and *indica* line R3-166 were selected from the BC_6_F_2_ populations (Fig. 1).

**Table 1:** Markers used for *Bph3* and *Bph27(t)* marker-assisted selection in this study

Mark name	Forward primer (5′–3′)	Reverse primer (5′–3′)
RH078	GTAAAAAAGTTGGAGTTGGC	GGCGAGTTGTGCTGTTG
RH7	CAGGTTTGGTTGAAGGGTCT	GAACTATGGCTCCACTGGTCTA
Q52	GCAAAGTACAAAACTAGCACA	TCAGTAAACTCACGAATAAAGC
Q31	GTTCCCTCATACGGATAG	AGATTTGACAAGGCTTACT
Q58	CATGCTGAGACCAAATTACTACA	GGGGTGGGCAAAATAAGA
RM471	ACGCACAAGCAGATGATGAG	GGGAGAAGACGAATGTTTGC

A total of 321 and 218 evenly distributed and polymorphic markers were used to identify the background of the introgression lines R2256 and R3-166, respectively. The results showed that NJ3 background of R2256 have arrived at 97.7 % (Fig. 1c, e), and 93-11 background of R3-166 also have arrived at 99.5 % (Fig. 1f). Therefore, these results demonstrated that *Bph27(t)* have been successfully introduced into NJ3 and 93-11 through MAS.

### Evaluation of BPH resistance and other agronomic traits of introgression lines

**Fig. 2:**
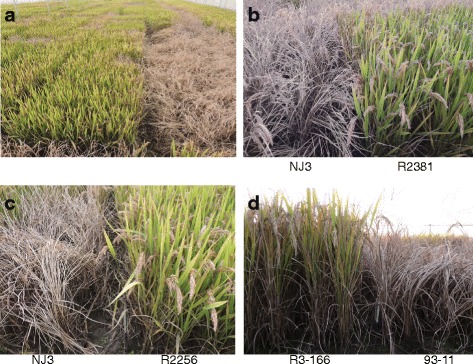
BPH resistance of *Bph27(t)*-containing introgression lines at mature stage in the field. **a** Overall view of the BPH resistance at mature stage; **b** *Bph3*-containing *japonica* near-isogenic line R2381; **c**, **d** *Bph27*-containing *japonica* and *indica* near-isogenic line R22256 and R3-166

To test whether the introgression of *Bph27(t)* can improve the BPH resistance of NJ3 and 93-11, we evaluated the BPH resistance of these introgression lines at the seedling stage under greenhouse conditions. While the recurrent parent NJ3 and 93-11 were completely dead, *Bph27(t)*-carrying lines R2256 and R3-152 showed no visible damage (Fig. 1). The BPH resistance evaluation was also conducted under artificial infestation conditions in the field at mature stage. The results showed that two introgression lines of *Bph27(t)* also displayed high resistance against BPH the same as that of *Bph3-*introgressing line R2381 at mature stage in the field (Fig. 2).

**Fig. 3:**
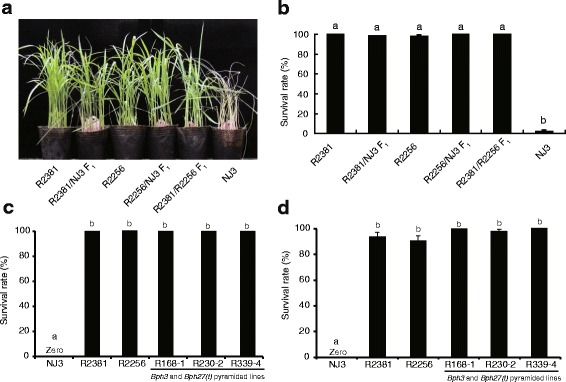
BPH resistance of *Bph3* and *Bph27(t) *homogenous and heterozygous genotypes (**a** and **b**), and two gene pyramided lines at seedling stage one-week and 3-weeks after infested with BPH (**c** and **d**). Letters indicate a significant difference at *P* < 0.01 by the Student’s *t*-test

To evaluate the resistance level of heterozygous *Bph27(t)* and potential application in hybrid rice breeding, R2381 and R2256 were separately backcrossed with NJ3 and intercrossed simultaneously. When the NJ3 plants nearly completely died, R2381/NJ3 F_1_, R2256/NJ3 F_1_ and R2381/R2256 F_1_ showed invisible damaged symptom (Fig. 3a, b). Similarly to *Bph3*, the results showed that *Bph27(t)* also is a dominant gene for BPH resistance.

**Fig. 4:**
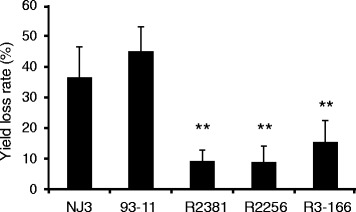
Actual yield loss rate of NJ3, 93-11, R2381, R2256 and R3-166 under BPH-infested conditions. Error bars indicate s.e.m. of the mean by the Student’s t-test (** *P* < 0.01)

The plants were harvested after ripening and the yield components including number of effective panicle and grains per panicle, seed setting rate and 1000-grain weight were examined (Table 2). Compared with controls, seed setting rate and 1000-grain weight of NJ3 infested with BPH were significantly reduced in both seasons. In contrast, the above components of introgression lines containing either *Bph27(t)* or *Bph3* were rarely influenced by BPH (Table 2). Upon infestation with BPH in 2014, the actual yield of NJ3 and 93-11 were reduced by 34.9 % and 43.6 %, respectively, but BPH caused little damage on R2381, R2256 and R3-166 (Fig. 4). Therefore, introducing *Bph27(t)* could significantly improve the BPH resistance and reduced the yield losses caused by BPH.

**Table 2:** Comparison analysis of the damage caused by BPH on recurrent parent and introgression lines

Lines	2013						2014				
		EPP	GPP	SSR (%)	TGW (g)	TYP (g)	EPP	GPP	SSR (%)	TGW (g)	TYP (g)
NJ3	Non-infested	10.07 ± 0.06	101.92 ± 1.17	95.67 ± 0.46	24.15 ± 0.07	23.72 ± 0.27	10.03 ± 0.02	101.23 ± 1.21	96.12 ± 0.22	24.27 ± 0.1	23.69 ± 0.89
	Infested	10.07 ± 0.07	102.14 ± 1.23	87.67 ± 1.61**	21.04 ± 0.23**	18.97 ± 0.28**	10.03 ± 0.09	104.38 ± 0.83	65.23 ± 1.74**	20.16 ± 0.33**	13.77 ± 0.67**
R2381	Non-infested	9.85 ± 0.15	99.27 ± 1.03	92.54 ± 0.15	24.14 ± 0.24	21.84 ± 0.22	9.91 ± 0.05	98.21 ± 0.98	92.54 ± 0.15	24.23 ± 0.11	21.82 ± 0.32
	Infested	9.79 ± 0.15	102.83 ± 5.92	88.59 ± 1.52	23.65 ± 0.25	21.09 ± 0.21	9.89 ± 0.16	101.36 ± 4.63	89.13 ± 2.24	22.88 ± 0.52	20.44 ± 1.89
R2256	Non-infested	9.85 ± 0.08	101.97 ± 2.04	90.94 ± 1.82	24.72 ± 0.16	22.08 ± 0.23	10.05 ± 0.05	92.68 ± 1.96	93.01 ± 2.02	24.52 ± 0.13	21.24 ± 0.97
	Infested	10.31 ± 0.21	102.83 ± 5.82	86.72 ± 0.37*	23.18 ± 0.26	21.08 ± 0.11	10.11 ± 0.19	94.96 ± 3.1	89.1 ± 1.87*	23.62 ± 0.14	20.2 ± 1.12

Number of effective panicle per plant (EPP), Number of grains per panicle (GPP), Seed setting rate (SSR), 1000-Grain Weight (TGW) and Theoretical yield per plant (TYP) of NJ3, R2381 and R2256 were measured under normal and BPH-infested conditions (*P < 0.05, ** *P* < 0.01)

### Pyramiding of *Bph3* and *Bph27(t)*

To pyramid the two BPH resistance genes and develop more durable BPH resistance rice, the plants with the two BPH resistance genes were screened from F_2_ population derived from R2381/R2256 F_1_, then *Bph3* and *Bph27(t)* pyramided lines were selected. Among them, eight lines with both homologous *Bph3* and *Bph27(t)* were subsequently evaluated for the BPH resistance. NJ3 were completely dead about 7 days after infestation with BPH, all three lines with zero mortality rate displayed high BPH resistance (Fig. 3c). When about 3-weeks after infestation, the survival rate of R2381 and R2256 arrived at about 5.1 % and 7.3 %, respectively, but the pyramided lines also showed invisible damaged symptom (Fig. 3d). These results indicate that *Bph3* and *Bph27(t)* pyramided lines showed higher resistance than single gene introgression lines.

## Discussion

Resistant cultivars with single resistance gene are currently hampered by the rapid breakdown of BPH resistance (Cohen et al. 1997; Alam and Cohen 1998). Thus, there is an urgent need to use more effective BPH resistance genes or pyramiding different resistance genes to develop more durable resistant rice cultivars. For example, *Bph14* and *Bph15* pyramided lines showed higher resistance than *Bph14* or *Bph15* single introgression lines (Hu et al. 2012). Pyramiding *Bph6* and *Bph12* line also showed significant additive effect against BPH compared with introgression of single gene (Qiu et al. 2012). These studies prove that pyramiding two or more BPH resistant genes is an efficient strategy to develop higher and durable BPH resistance cultivars (Chen et al. 2000; Tu et al. 2000; Hittalmani et al. 2000)*.* Compared with previous used genes, such as *Bph1* and *Bph2*, *Bph3* performs more broad spectrum and durable resistance (Cruz et al. 2011). *Bph27(t)* is also a high BPH resistance gene. The pyramiding of *Bph3* and *Bph27(t)* will provide more useful material for breeding durable BPH resistant cultivars. *Bph3* and *Bph27(t)* were linked on rice chromosome 4, moreover, RH and Ba the two donor parents come from Sri Lanka, the Indel markers used for selection of *Bph3* and *Bph27(t)* showed non-polymorphic between RH and Ba, so it is difficult to distinguish the two genes in the process of pyramiding the two genes. In order to pyramid *Bph3* and *Bph27(t)*, single-gene introgression lines without each other locus were developed firstly, then, the two resistance genes were pyramided by intercrossing with these single-gene introgressed lines through MAS into commercial *japonica* rice cultivar. The two genes pyramided lines displayed higher resistance than those lines with single resistance gene. The development of two genes pyramided lines provides innovative resources for breeding of durable BPH-resistant rice cultivars

The BPH resistance level of heterozygous genotype is important for hybrid improvement. Some BPH genes display partial dominant resistance, and their heterozygotes show moderately resistance or even susceptible to BPH. If these genes will be integrated in hybrid improvement, they have to be separately introgressed into two parent cultivars to guarantee the resulting hybrids carry multiple and complementary resistance genes. Hybrids between *Bph3* or *Bph27(t)* introgression lines and NJ3 showed similar BPH resistance with homologous *Bph3* or *Bph27(t)* introgressed lines. In accordance with previous studies (Lakshminarayana and Khush 1977; Ikeda and Kaneda 1981; Nemoto et al. 1989), these results indicate both genes are dominant BPH resistance genes. This made them great potential in future hybrid improvement.

‘Linkage Drag’ is a common phenomenon in disease and insect resistance breeding program (Lewis et al. 2007; Liu et al. 2009; Young and Tanksley 1989). RH and Ba the two donor parents are Sri Lanka landrace varieties, exhibited large genetic divergence with *japonica* rice cultivars. After six times backcross with NJ3, we also found the partial anthers indehiscence of single gene introgression lines resulted in the reduction of spikelet fertility compared with that of NJ3 under BPH non-infestation condition (Table 2). We detected the genetic background of the *japonica* ingression line and found that a large fragment of chromosome 4 from Ba was introduced into the recipient parent (Fig. 2). Previously several sterile loci in *indica*-*japonica* cross were found on the short arm of chromosome 4, such as *S9* and *S28* (Sobrizal et al. 2002; Zhao et al. 2006). We speculated the lower spikelet fertility may be caused by these sterile loci. In order to break the linkage drag, we tried to continuously backcross with NJ3. Unexpectedly, our efforts failed due to serious segregation distortion. The break of linkage drag would depend on a larger population in the future study.

Previous studies usually introgressed BPH resistance genes into *indica* rice cultivars. However, BPH has rose up as major destructive insect throughout *Japonica* varieties grown area. The absence of BPH resistance *Japonica* cultivars will put rice production in danger. In present study, we successfully introduce *Bph27(t)* into commercial *Japonica* variety NJ3 based on MAS, furtherly pyramided *Bph27(t)* with *Bph3* in *Japonica* variety. These introgression lines can act as excellent ‘base materials’ and will have wide applications in breeding durable BPH resistant varieties.

## Conclusions

We introgressed single or two BPH resistance genes into two elite rice cultivars using MAS. The introgression of BPH resistance genes significantly improved the BPH resistance level and reduced the yield loss caused by BPH. The development of BPH resistance lines has a promising future in molecular breeding of durable BPH-resistant rice cultivars.

## Methods

### Plant materials and BPH population

Balamawee (Ba) with *Bph27(t)* were used as BPH-resistant donor parent. *Japonica* cultivar (cv.) Ningjing3 (NJ3) and *indica* cultivar (cv.) 93-11 were used as recurrent parent. R2381, a *Bph3*-carrying line was selected from a BC_6_F_2_ population by backcross between donor parent RH and recurrent parent NJ3 (Liu et al. 2015). BPH population was originally collected from rice fields in Nanjing, China and maintained on TN1 plant under greenhouse condition at Nanjing Agricultural University.

### Evaluation of BPH resistance

A seedling bulk test was conducted to study plant reaction to BPH feeding according to the standard evaluation systems of IRRI with minor modifications (IRRI, 1988). To ensure all seedlings were at the same growth stage for BPH infestation, seeds were pre-germinated. About 30 seeds from individual plant were sown in a 10 cm-diameter plastic pot with a hole at the bottom. Seven days after sowing, seedlings were thinned to 25 plants per pot. At the second-leaf stage, the seedlings were infested with 2nd to 3rd instar BPH nymphs at 10 insects per seedling. When all the TN1 plants were dead, the seedling mortality of other cultivars or lines was recorded. Three replicates were used for each cultivar or line.

For evaluation of BPH resistance in the field, in the summer of 2013 and 2014 at Nanjing, the introgression lines and recurrent parents were alternatively grown in a sealed field with Nylon net with three replicate plots at a spacing of 16.7 cm × 20 cm. Each plot consisted of 10 rows with 10 plants per row. About 100 BPHs per plant were infested at the booting stage. Another three replicate plots were cultivated in other field as the controls. Water and fertilizer were managed regularly. Ten individuals were taken for measurements of agronomic traits, including productive panicles per plant, total grains per panicle, seed setting rate, and 1000-grain weight. In 2014, the actual yield of each plots were measured and the yield loss rate were calculated by the following formula:

Yield loss rate (%) = 1- Yield under BPH infestation condition/Yield under normal condition

### DNA extraction and non-denaturing PAGE

Plant DNA was extracted from fresh rice leaves using the CTAB method (Murray and Thomson 1980). The extracted DNA was dissolved in TE buffer for further analysis. Amplified products were separated in 8 % non-denaturing PAGE and visualized with silver staining (Sanguinetti et al. 1994).

### Introgression of *Bph27(t)* and pyramiding *Bph27(t)* and *Bph3* through MAS

The procedure of introgression and pyramiding *Bph27(t)* and *Bph3* was as described in Additional file 1: Figure S1. To introgress *Bph27(t)* to *Japonica* cultivar NJ3, we firstly crossed Ba with NJ3 and got hybrid F_1_, then, F_1_ was continuously backcrossed with NJ3 for 6 times. In each generation, two Indel markers Q52 and Q31 were used to select positive progenies. BC_6_F_1_ were self-crossed to produce BC_6_F_2_. The homozygous plants at *Bph27(t)* in BC_6_F_2_ were selected. Meanwhile, the same procedure was conducted to introgress *Bph27(t)* to *indica* cultivar 93-11, except that markers were replaced by RM471 and Q58 (Table 1).

Then, *Bph27(t)* and *Bph3* introgressed *Japonica* lines R2256 and R2381 were crossed to pyramid the two BPH resistance genes.

## Additional file

Additional file 1: Figure S1. The procedure of introgression and pyramiding *Bph27(t)* and *Bph3*. (PDF 338 kb)
