# Supplementary figures and images for: Marker assisted pyramiding of two brown planthopper resistance genes, Bph3 and Bph27 (t), into elite rice Cultivars
Source: Rice (N Y). 2016 May 31;9:27. doi: 10.1186/s12284-016-0096-3 (PMC4887400; doi:10.1186/s12284-016-0096-3)

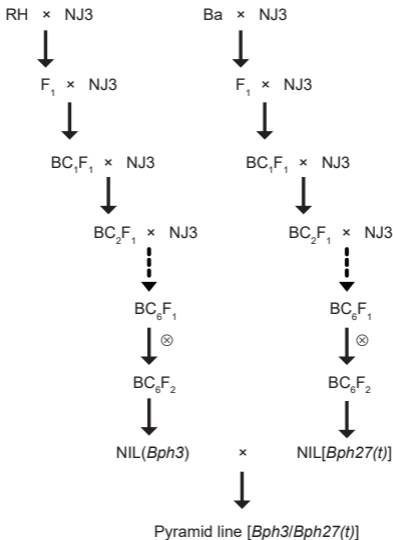

**Figure S1** The procedure of introgression and pyramiding *Bph27(t)* and *Bph3*

Supplement: Additional file 1: Figure S1. — The procedure of introgression and pyramiding Bph27(t) and Bph3. (PDF 338 kb) [file 12284_2016_96_MOESM1_ESM.pdf]
